# Anti-TNF inhibits the Airways neutrophilic inflammation induced by inhaled endotoxin in human

**DOI:** 10.1186/2050-6511-15-60

**Published:** 2014-11-03

**Authors:** Olivier Michel, Phong Huy Duc Dinh, Virginie Doyen, Francis Corazza

**Affiliations:** Clinic of Allergology and Immunology, CHU Brugmann (Université Libre de Bruxelles - ULB), 4 pl Van Gehuchten, B -1020 Brussels, Belgium; Laboratory of Immunology, CHU Brugmann (Université Libre de Bruxelles - ULB), Brussels, Belgium; Department of Immunology, Pham Ngoc Thach University of Medicine (PNTU), Ho Chi Minh, Vietnam

**Keywords:** Endotoxin inhalation, Neutrophilic inflammation, Corticosteroids, Anti-TNF

## Abstract

**Background:**

Inhaled endotoxin induces airways’neutrophilia, in human. TNF-a being a key cytokine in the response to endotoxin, the effect of anti-TNF on the endotoxin-induced neutrophilic response was evaluated among healthy volunteers.

**Methods:**

Among a population of 30 healthy subjects, an induced-sputum was collected 2 weeks before, and 24 hours after an inhalation of 20 mcg endotoxin (E coli 026:B6). Then, the subjects were randomized into 3 parallel groups treated with control, oral methylprednisolone 20 mg/day during 7 days or anti-TNF (adalimumab, Humira®, Abbott) 40 mg SC. One week later, an induced-sputum was sampled, 24 hours after an inhalation of endotoxin.

**Results:**

After endotoxin inhalation, the number of total cells, neutrophils and macrophages was significantly increased (p <0.001). Compared to the response to endotoxin among the control group, anti-TNF inhibited the endotoxin-induced neutrophil influx, both in relative (51.3 (±6.4)% versus 26.2 (±5.3)%, p <0.002) and in absolute values (1321 (443–3935) cells/mcL versus 247 (68–906) cells/mcL, p <0.02). The endotoxin-induced neutrophilic response was not significantly modified among the control group and oral corticosteroid group.

**Conclusions:**

While oral corticosteroid had no effect, anti-TNF inhibited the neutrophil influx in sputum, induced by inhalation of endotoxin, in human subject. The endotoxin model could be an early predictor of clinical efficacy of novel therapeutics.

**Trial registration:**

ClinicalTrials.gov NCT02252809 (EudraCT2008-005526-37)

## Background

Over one bilion people through the World suffer from chronic respiratory diseases (CRD), mainly chronic obstructive pulmonary diseases (COPD) and asthma [1]. Currently there is no satisfactory treatment for COPD and severe asthma. Airways’ neutrophilic inflammation is a risk factor of severity of several CRD. The number of neutrophils in sputum correlates with the severity [2] and accelerated decrease of FEV1 [3] in COPD and with severe exacerbations in asthma [4]. Neither oral corticosteroids (CS), nor a high dose inhaled CS has an effect on the airways’ neutrophilic inflammation in COPD [5, 6], and neutrophilic exacerbations of asthma are refractory to increasing the dose of inhaled corticosteroids [7]. Through the activation of NF-kB, TNF-a induces the IL-8 chemokine that is a chemoattractant for the neutrophils. Consistently, some studies reported that the concentrations of TNF-a and its soluble receptor are raised in the sputum of COPD patients [8]. The lack of anti-inflammatory effects of CS in COPD could be related to the reduction in recruitment of histone desacetylase-2 by CS, resulting in the absence of control of NFkB transcription, leading to expression of cytokines such as TNF-a and IL-8 [9]. Thus, TNF-a appears to participate to the mechanism of airways neutrophilic inflammation in COPD and severe asthma.

The endotoxin-induced airways’ inflammation mimicks several aspects of acute exacerbation of COPD [10]. This neutrophilic inflammation is not modified by oral prednisolone [11]. In an ex-vivo model, using endotoxin exposure of lung tissue from COPD, TNF was the initial cytokine and was predicitive for the following release of IL-6, CXCL8 and IL-10. It was inhibited by the neutralisation of the TNFα [12]. The concentration of TNF in the bronchoalveolar lavage was significantly increased during the early phase [2 hours] after bronchial endotoxin instillation in human [13]. Recently the involvement of NF-kB activation in the neutrophilic response to inhaled endotoxin has been reported among smokers [14].

Since TNF-a seems to be a key cytokine in endotoxin-induced neutrophilic inflammation, the current study evaluated the inhibiting effect of anti-TNF on the neutrophilic response among healthy volunters exposed to inhaled endotoxin.

## Methods

### Subjects

A population of 49 healthy, male and female, non-smoker volunteers (age 18 to 50 years) was screened, after a written informed consent was obtained from each subject. They were excluded if they used drugs within 2 weeks or over-the counter medication.

### Study design

**Figure 1:**
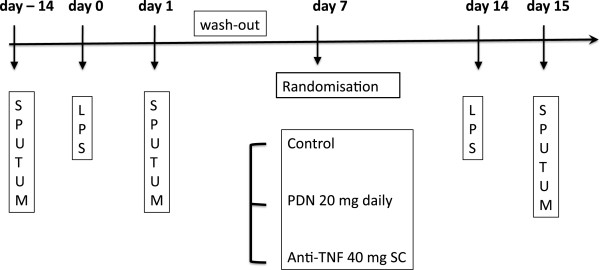
**The design of the study.**

During the screening phase, an induced-sputum was collected 2 weeks before, and 24 hours after an inhalation of 20 mcg endotoxin. On day 1, among the 49 healthy volunteers, 40 were selected after having produced a valid sputum (defined as a 80% or more viability, with less than 50% squamous cells, and less than 70% neutrophils). A significant inflammatory response to inhaled endotoxin was defined as an increase of 10% or more of the absolute count of neutrophils in the sputum. By doing so, 30 subjects were included (mean age: 31.0 (28 – 34) years; females/males: 16/14) (Figure 1).

After a wash-out period of 7 days, they were randomised into 3 open parallel groups: control or treated with 20 mg oral prednisolone (Medrol®, Pfizer-Upjohn) once daily for 7 days (PDN) or a single sub-cutaneous anti-TNF antibody, 40 mg adalimumab (Humira®, Abbott) on day 1. On day 14, a challenge test with inhaled endotoxin was performed in each subject and an induced-sputum was obtained 24 hours later. A clinical follow-up visit was performed after 5 weeks.

### Induced sputum

Hypertonic sterile saline (5%) was nebulized for 30 minutes with an ultrasonic nebulizer (Fisoneb; Karapharm, Marseille, France); subjects rinsed their mouth with water every 10 minutes and tried to cough sputum directly into a sterile plastic box. After selection of all portions of sputum as free as possible of saliva, the plugs were weighed, mixed with 4 volumes of dithiotreitol 0.1% (Sputolysin; Behring Diagnostics, Somerville, NJ), homogenised and rocked for 15 min. before adding 4 volumes of Dulbecco’s PBS. After filtration and centrifugation (15 minutes at 800 g) the supernatant was frozen at -80°C while the pelleted cells were resuspended in PBS. The number of total cells was measured with a Thoma's hemocytometer. The cell viability was assessed by the Trypan blue method. A slide was prepared by centrifugation (Cytospin, Shandon Inc, Pittsburgh, PA) and stained with May-Grünwald-Giemsa. The differential cells were counted on 400 cells.

### Endotoxin challenge tests

The procedure of endotoxin challenge has been previously reported [15]: briefly 20 μg of a suspension of lipopolysaccharide (LPS Escherichia coli 026:B6 from Sigma Chemical, St Louis, MO -ref L-2654), the active derivative of endotoxin, was administered by a dosimeter Mefar MB3 (Mefar, Brescia, Italy). The dose of inhaled endotoxin corresponded to 17 inhalations of a calibrated aerosol of 6 mcL/inhalation containing a solution of 0.2 mg/mL endotoxin. Outputs was checked by weighing the nebulizer containing 2 ml of sterile normal saline before and after 10 actuations [16]. The endotoxin dose was selected according to data published on the dose–response relationship to inhaled endotoxin [17]. The objective was to cause only minimal systemic responses, though with a significant but sub-maximal inflammatory responses in the lung, to allow prednisolone and/or adalimumab to significantly reduce these responses. At the end of the procedure, the volunteers were instructed to rinse their mouth to eliminate residual endotoxin trapped on the oral mucosa. Symptoms, oral temperature, forced vital capacity (FVC), forced expiratory volume in 1 second (FEV1) and the FEV1/FVC were recorded before and hourly after endotoxin.

### Good clinical practice

This study was conducted according to with Good Clinical Practice Guidelines of the International Conference on Harmonisation. The study was registered in the non-public database of all drugs trials in the European Community (EudraCT: 2008-005526-37), as an exploratory phase study, in 2008, and then in the public database form the ClinicalTrials.gov NCT02252809. It was approved by the Ethics Committee of the CHU Brugmann (decision number CE2008/49) and the competent authorities in Belgium. Written informed consent was obtained in each subject. The Clinical Research Unit of the Institution was responsible for study coordination.

### Statistics

The results were expressed as mean or geometric mean ±95% confidence interval. The absolute values of the cells were Log transformed. Repeatability of the response to LPS was assessed, among the control group, by plotting the differences between repeated measurements against the mean of the repeated measures, and testing whether the mean differences was significantly different from 0 (method of Bland and Altman) [18]. ANOVA was used to compare change from baseline among the three groups (control, PDN, anti-TNF), followed by paired t-tests between each active treatments and the control group. P values smaller than 0.05 were considered statistically significant.

## Results

The demographics of the population is shown in Table 1. There was no significant difference among the 3 randomised groups for age, sex ratio, sputum characterictics at the basal state.

**Table 1:** **Demography and sputum characteristics at the basal state in the whole population and among each randomized group**

	Whole population	Control	PDN	Anti-TNF
Subjects (n)	30	10	10	10
Sex (F/M)	16/14*	4/6	5/5	7/3
Age (years, 95% CI)^†^	31 (28–34)	29 (23–34)	33 (27–40)	31 (25–37)
Weight of sputum plugs (mg, 95% CI)^‡^	469 (389–553)	564 (378–750)	422 (309–535)	433 (260–606)
Cells viability (%, 95% CI)^†^	80.4 (76.0-84.7)	80.7 (73.7-87.6)	85.6 (78.3-92.8)	74.8 (65.5-84.2)
Total cells (cells/μL, 95% CI)^‡^	2280 (1496–3467)	4083 (1749–9506)	2138 (933–4909)	1356 (764–2404)

^†^arithmetic means; ^‡^geometric means.

*age, sputum characteristics were not significantly different among female (F) compared to male (M).

Except a slight headache among 5 subjects, there was no significant symptom or change in lung function. The temperature increased slighly from 36.3 (36.0-36.6) immediately before LPS to 36.3 (36.0-36.5) (p = NS), 36.3 (36.2-36.7) (p = NS), 36.4 (36.1-36.6) (p = NS) , 36.5 (26.2-36.8) (p <0.05), at 2, 4, 8 and 24 hours after inhaled LPS, respectively. There was no significant drug effect on the symptoms and/or the temperature.

**Figure 2:**
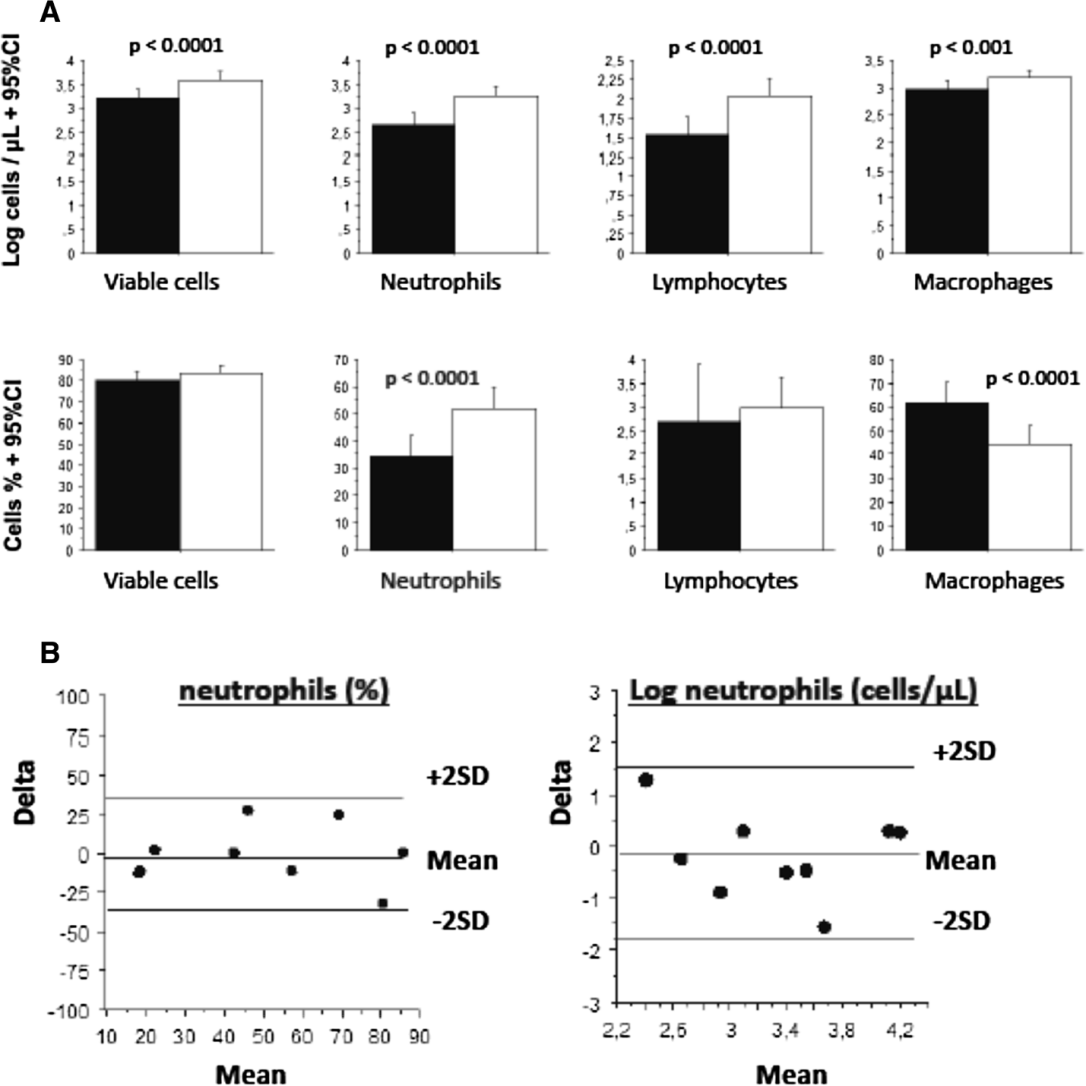
**The change of the sputum cells count after LPS inhalation compared to saline inhalation. A**. Sputum cells count before and 24 hours after inhalation of 20 mcg endotoxin during the screening phase, among the 30 included subjects. The black bar indicates the cells counts after saline, the white bar after endotoxin inhalation. Data (in % or log absolute value) are expressed as means +95% CI. Statistics: paired t-tests (n = 30). **B**. Assesment of repeatability of the LPS induced neutrophils (% and absolute values). The differences against the means of the neutrophils counts, after repeated inhaled LPS at day 1 and day 14, among the control group. Limits of agreement are the mean ±2 SD.

Among the 30 subjects, during the screening phase, the endotoxin inhalation induced a significant rise of the geometric means of total viable cells (p <0.0001), neutrophils (p <0.0001), macrophages (p <0.001), and lymphocytes (p <0.0001) (Figure 2A). The arithmetic means of the percentage of neutrophils increased from 35.0 (26.9 – 43.1)% to 52.4 (44.6 – 60.3)%, (p <0.0001), while there was a decrease of macrophages from 60.7 (52.2 – 62.2)% to 43.8 (35.9 – 51.8)%, (p <0.0001) (Figure 2A). Among the subjects of the control group, the neutrophilic (%) response correlated significantly between the 2 endotoxin challenges (i.e. the endotoxin challenge before and after randomisation, r = 0.78; p <0.02), suggesting that the response was reproducible. The intra-subject repeatability of the method was evaluated in the control group, by comparing the neutrophilic response on day 1 and day 14 (Figure 2B). The Bland and Altman analysis showed that the measurements of percentage and absolute values of neutrophils were not statistically different between day 1 and day 14 (t-test = -0.179 and -0.585, respectively).

**Figure 3:**
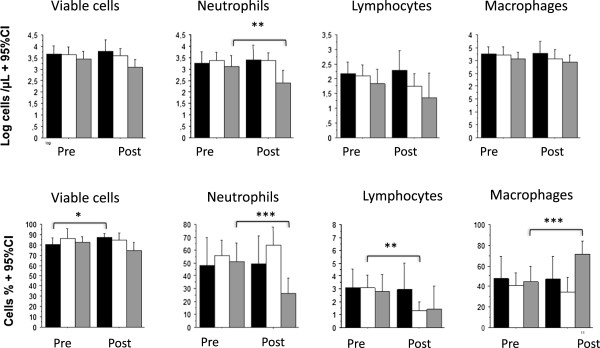
**Comparisons of LPS-induced cell counts before and after treatment.** The black bar indicates the cells counts after control, the white bar after methylprednisolone and the gray bar after anti-TNF. Data are expressed as means +95% CI. Statistics: paired t-tests. *p <0.05, **p <0.02, ***p <0.002.

Anti-TNF inhibited the neutrophil influx both in relative (51.3 (36.8 - 65.8)% versus 26.2 (14.1 – 38.2)% , p <0.002), and in absolute value (1321 (443–3935) cells/mcL versus 247 (68–906) cells/mcL, p <0.02) (Figure 3). While anti-TNF increased the percentage of macrophages (44.7(29.8 – 59.6)% versus 71.3 (58.4 – 84.1)% ; p <0.002), it had no significant effect on the absolute count of macrophages (1180 (661–2089) cells/mcL versus 873 (457–1660) cells/mcL; p = NS) (Figure 3). Oral corticosteroid had no significant effect on the neutrophils and macrophages response to endotoxin, while it inhibited significantly the percentage but not the absolute count of lymphocytes (Figure 3). The data of the endotoxin-induced cells count response in sputum before and after a treatment are shown in Table 2.

**Table 2:** **The endotoxin-induced cells count response in sputum before and after a treatment with control, oralsteroids and anti-TNF**

Parameters	Total	Control	PDN	p value ^‡^	Anti-TNF	p value ^‡^
		.(1)	.(2)	(1) vs (2)	.(3)	(1) vs (3)
n	30	9	10		10	
**Total viable cells (cells/μL)**						
Before LPS	1811 (1191–2748)	2716 (1132–6741)	1820 (805–4111)		1000 (607–1648)	
After LPS	4188 2754–6368)	4570 (2004–10399)	4447 (2060–9571)		2884 (1352–6152)	
After LPS + treatment		6039 1883–19408)	3963 (2004–7834)	NS	1261 (591–2691)	0.052
**p value** ^**†**^	< 0.0001					
**Neutrophils (cells/μL)**						
Before	520 (299)906)	789 (229–2710)	561 (207–1517)		244 (120–498)	
After LPS	1954 (1135–3357)	1795 (560–5768)	2387 (1019–5598)		1321 (443–3935)	
After LPS + treatment		2594 (614–10990)	2313 (1084–5284)	NS	247 (68–906)	< 0.02
**p value** ^**†**^	p <0.0001					
**Macrophages (cells/μL)**						
Before	993 (681–1449)	1438 (692–2985)	914 (395–2113)		656 (396–1089)	
After LPS	1618 (1138–2296)	1786 (939–3396)	1644 (807–3350)		1180 (661–2089)	
After LPS + treatment		1892 (647–5508)	1185 (528–2654)	NS	873 (457–1660)	NS
**p value** ^**†**^	p <0.001					
**Lymphocytes (cells/μL)**						
Before	39 (23–65)	65 (29–147)	33 (12–91)		21 (8–54)	
After LPS	119 (71–201)	155 (64–378)	125 (54–299)		67 (20–219)	
After LPS + treatment		198 (42–929)	54(18–156)	NS	22 (3–159)	NS
**p value** ^**†**^	p <0.0001					
**Neutrophils (%)**						
Before	35 .0 (26.9-43.1)	36.8 (18.3-55.3)	37.3 19.0-55.3)		28.2 (18.2-38.1)	
After LPS	52.4 (44.6-60.3)	48.4 (27.1-69.8)	55.9 (44.0-67.8)		51.3 (36.8-65.8)	
After LPS + treatment		49.6 (28.2-70.9)	63.8 (49.8-77.8)	NS	26.2 (14.1-38.2)	< 0.002
**p value** ^**†**^	p <0.0001					
**Macrophages (%)**						
Before	60.7 (52.2-62.2)	58.6 (39.8-77.5)	59.5 (39.5-79.5)		67.1 (57.2-77.0)	
After LPS	43.8 (35.9-51.8)	47.8 (26.2-69.3)	40.8 (28.7-52.8)		44.7 (29.8-59.6)	
After LPS + treatment		46.9 (24.5-69.3)	34.6 23.4-48.8)	< 0.002	71.3 (58.4-84.1)	NS
**p value** ^**†**^	p <0.0001					
**Lymphocytes (%)**						
Before	2.7 (1.5-3.9)	2.1 (0.8-3.4)	2.7 (0.6-4.8)		3.3 (0.1-6.3)	
After LPS	3.0 (2.3-3.6)	3.1 (1.6-4.6)	3.1 (2.4-4.1)		2.8 (1.4-4.1)	
After LPS + treatment		2.9 (0.9-5.0)	1.3 (0.6-2.0)	NS	1.5 (0.3-3.2)	0.052
**p value** ^**†**^	NS					

The % and absolute values are expressed as arithmetic or geometric means, respectively (95% Confidence Interval). PDN, methylprednisolone.

^‡^Unpaired t-tests were used to compare the changes among control (1) with the changes after prednisolone (2) or anti-TNF (3).

^†^Paired t-test to compare the cell counts before and after LPS, among the whole population.

**Figure 4:**
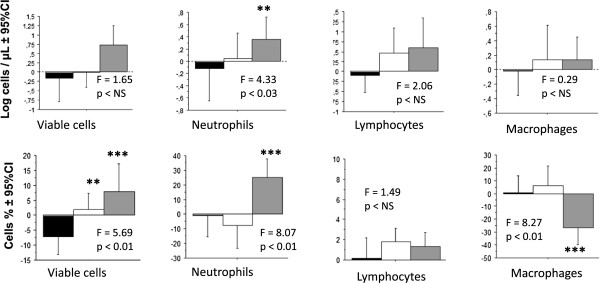
**Comparison of the change of sputum cell counts after a treatment with control, prednisolone or anti-TNF.** The red bar indicates the change after treating with anti-TNF, the green bar is the change after methylprednisolone and the white bar is control. Data are expressed as means +95% CI. ANOVA among the 3 treatments was applied followed by comparisons among the treatments; paired t-test applied when F-test is significant. Statistics: paired t-tests. **p <0.02, ***p <0.002.

The endotoxin-induced changes of the cell counts of the control group were compared to the changes after treatment with corticosteroids or anti-TNF (Figure 4, ANOVA for repeated measurements). The F-tests were significant for PMN % (F^2^_26_ = 8.07, p <0.01), macrophages % (F^2^_27_ = 8.27, p <0.01), viability % (F^2^_26_ = 5.69, p <0.01), and Log PMN (F^2^_26_ = 4.33 , p <0.03), but not for Log macrophages, Log lymphocytes and lymphocytes %.

**Figure 5:**
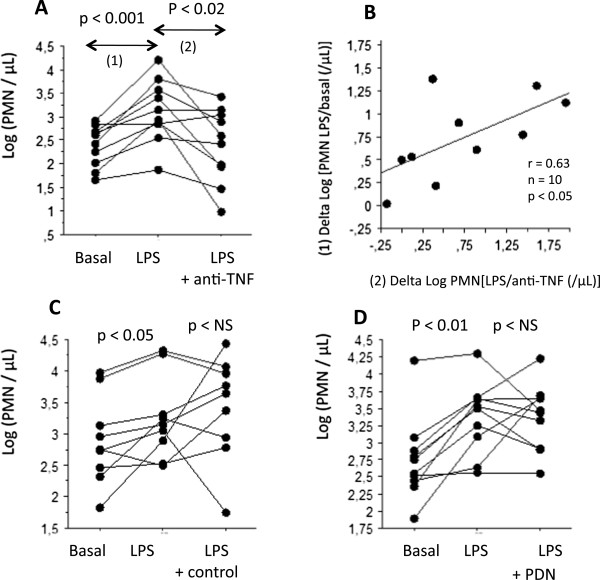
**The individual values of the PMN response after endotoxin. A**. The individual values of Log count of neutrophils in sputum at the basal state, after endotoxin inhalation(LPS) and after LPS with a previous treatment with anti-TNF (LPS + anti-TNF). **B**. The relationship between the rise of the Log count of neutrophils in sputum after endotoxin (LPS) (vertical axis) with the inhibiting effect of anti-TNF on the LPS response (horizontal axis). **C**. The individual values of Log count of neutrophils in sputum at the basal state, after endotoxin inhalation(LPS) and after LPS among the control group. **D**. The individual values of Log count of neutrophils in sputum at the basal state, after endotoxin inhalation(LPS) and after LPS with a previous treatment with oral steroids (PDN).

The amplitude of the neutrophil response to endotoxin, expressed in absolute values was variable among the subjects and not related with the neutrophil count at the basal state (Figure 5A). Anti-TNF totally inhibited the endotoxin-induced rise of neutrophils, in each subject (exept one). The rise of the neutrophilic count after endotoxin (Figure 5B) was significantly related to the amplitude of the anti-TNF blocking effect, suggesting that anti-TNF was mainly active on the endotoxin-induced change in neutrophils but not, on the airways neutrophils count at the basal state. There was no significant change of the amplitude of the neutrophil response to endotoxin in both the control groups and the oral steroids treated subjects (Figure 5C and D).

## Discussion

The study shown that a pretreatment with anti-TNF inhibited the endotoxin-induced neutrophil influx in induced sputum, among healthy subjects. Conversely, oral corticosteroid had no effect on the endotoxin induced inflammation.

In human, a bronchial instillation of endotoxin induced an early phase reaction, occuring after 2 hours and characterized by a local increase in neutrophils and cytokines, such as TNF-a, IL1-b, IL-6 and IL-8 [13]; TNF-a increased more than 300 fold, compared to the control [13]. It was followed by a later phase (24–48 hours) characterized by the presence of neutrophils, macrophages, macrophages and lymphocytes [13, 19], recovering within 7 days [19]. In ex vivo lung tissue, stimulated with endotoxin (100 ng/ml), TNF-a was the initial cytokine, expressed by the macrophages and mastocytes as early as 1 hour, rising at 2 and 4 hours and peaking at 6 hours and it was also predictive for the following release of cytokines, after endotoxin exposure [12]. Since TNF-a is a key cytokine in endotoxin-induced airway’ inflammation, we hypothesised that anti-TNF could attenuate the endotoxin induced airways’ neutrophilia.

The current data confirmed the airways’ neutrophilic response in absolute and relative value, 24 hours after an inhalation of 20 mcg endotoxin. Firstly, we evaluated the repeatability of the PMN response. It is well known that the amplitude of the neutrophilic response to inhaled endotoxin is highly variable between subjects [20], as also confirmed by the present results. Nevertheless,in the current study, among the 10 subjects submitted to repeated endotoxin challenges, at 14 days interval, the intra-subject repeatability of the sputum neutrophilia was significant, consistently with recent data [14, 21, 22].

Secondly, we investigated the effect of oral corticosteroids on the endotoxin-induced airways’ neutrophilia. A 7 days pretreatment with methylprednisolone 20 mg daily had no significant effect. In a previous study the same drug regiment did not have an effect on the response to 50 mcg LPS, a dose that has been associated with clinical symptoms [11]. It was thought that exposure to a subclinical (and consequently sub-maximal) dose of LPS could be blocked by oral steroids but it was not confirmed by the current data. We can not excluded that for a lower level of LPS exposure (such as 5 μg), an effect of oral corticosteroids on biomarkers of inflammation could be significant; though at this level of exposure, the response is inconsistent between subjects [14, 17].

Thirdly, we have shown that, in contrast to corticosteroids, a pretreatment with anti-TNF blocked the neutrophilic response, both in relative and absolute values. In mirror with the decrease of the neutrophils, the percentage of macrophages increased, though the absolute number of macrophages remained unchanged after anti-TNF treatment. Interestingly the current data shown that anti-TNF had a blocking effect mainly on the rise of the neutrophils rather than the basal sputum neutrophilia. This suggests that anti-TNF could be active rather on neutrophilic exacerbations, than on the basal state.

Patients with refractory asthma have evidence of up-regulation of the TNF-α axis since they had increased expression of membrane-bound TNF-α, TNF-α receptor 1, and TNF-α–converting enzyme by peripheral-blood monocytes and a 10 weeks of treatment with the soluble TNF receptor, etanercept, was associated with a significant improve in non specific bronchial hyperresponsiveness, post bronchodilator FEV1 and asthma-related quality of life [23]. In moderate asthma, the anti-TNF infliximab had no effect on morning peak expiratory flow, though it reduced of more than 50% the number of moderate exacerbations [24]. A recent case series suggesting that anti-TNF may improve the condition of severe steroid-dependent refractory asthma, with frequent exacerbations and daily symptoms despite close repeated medical evaluation and maximal treatment including oral steroids [25]. In animal model of allergen sensitization, on the contrary to corticosteroids, anti-TNF does not modify the allergen ovalbumin-induced airways reaction. Though, when ovalbumin is mixed with endotoxin, anti-TNF significantly blocked the inflammatory reaction, suggesting that TNF may play a more prominent pathogenic role in patients with an environmental exposure to endotoxin [26].

The importance of TNF in severe corticoresistant asthma was also suggested by increased protein and gene expression in the airways [27]. The gene expression profiling in induced sputum, shown that upregulation of TNF was associated with neutrophilic asthma [28]. TNF-a is also believed to play a central role in the pathophysiology of COPD [29]. Since, on one side severe asthma and COPD are heterogeneous diseases with different phenotypes and endotypes and, on the other side the TNF inhibitor have blocking effect on endotoxin-induced airways’ neutrophilic inflammation, future studies could investigate what kind of patient can benefit from anti-TNF, in regard to their inflammatory sensitivity to endotoxin [30].

Other anti-inflammatory drugs have been evaluated on endotoxin induced airways’ inflammation in human. Salmeterol was shown to have a significant anti-inflammatory effect, even when a 100 μg dose of inhaled endotoxin was used [31]. It has been reported that neutrophilic inflammation induced by intra-nasal instillation was reduced by inhibition of CXCR2 (a chemokine receptor antagonist) [32]. Recently an oral CXCR2 antagonist inhibited the induced sputum inflammation, induced by inhaled endotoxin, among healthy volunteers [33]. Simvastatin inhibits inflammatory responses in vitro and in murine models of lung inflammation in vivo; a pretreatment with simvastatinreduced the lung neutrophilic response induced by LPS inhalation in human volunteers [34]. The PDE-4 inhibitors have been also evaluated on the LPS model. Roflumilast reduced the neutrophilic into the airways after segmental bronchial challenge with endotoxin [35] – however not confirmed in a recent study using GMP-grade LPS [21] -, while cilomilast had no effet on the endotoxin-induced sputum neutrophila [11]. Interestingly, the phase III development of cilomilast have been stopped due to a lack of efficacy, while roflumilast has received post-phase III market autorisation. Recently it has been reported that in human volunteers, a pretreatment with vitamin E decreased the neutrophilic airways’ response induced by endotoxin [36].

The data obtained with the endotoxin model among healthy subjects have been extrapolated to the COPD patients. Based on their own data, R Kitz et al. concluded that the endotoxin inflammation is a model to investigate the inflammatory response in human and to improve our understanding of the mechanism of chronic respiratory diseases [37]. Because inhalation of endotoxin induced inflammation mimicking several characteristics of COPD, Korsgen et al. considered that the endotoxin model in human could be used for initial human studies of novel COPD-drugs [38]. According to Aul et al., the endotoxin response could be a suitable model of bacterial exacerbations of COPD since the response is safe, reproducible and associated to translocation of the NF-kB subunit p65 in sputum cells [14]. In a brief review comparing the endotoxin model with ozone and rhinovirus challenges, the endotoxin is the model of choice for new drugs involving the TLR4 receptor, and NF-kB pathway [39]. Since the current data show that endotoxin inflammation is inhibited by adalimumab, a TNF inhibitor, this last could be used as a positive control, in future studies evaluating novel agents.

## Conclusions

While oral corticosteroid was not effective, the TNF inhibitor adalimumab blocks the endotoxin-induced neutrophilic airways’ inflammation.

Firstly, this endotoxin model could be used to understand the biological effects of compounds that inhibit the LPS induced NF-kB pathway and/or be a model of acute exacerbation of COPD and it could be an early predictor of clinical efficacy of novel therapeutics. Secondly, an anti-TNF treatment could be indicated in chronic respiratory diseases with acute neutrophilic airways’ exacerbations, in particular related to endotoxin sensitivity and/or environmental exposure.

## Abbreviations

COPD: Chronic obstructive pulmonary disease
CRD: Chronic respiratory diseases
CS: Corticosteroids
FEV1: Forced expiratory volume in one second
FVC: Forced vital capacity
LPS: Lipopolysaccharide
PDN: Prednisolone
PMN: Polymorphonuclear neutrophils
TNF: Tumor necrosis factor.
